# Research on the innovation of early warning mechanisms of major public health emergencies for poverty alleviation and marginal populations: a case study of Fujian Province

**DOI:** 10.3389/fpubh.2024.1474776

**Published:** 2024-12-11

**Authors:** Haitang Huang, Jianlun Teng, Qingshui Li

**Affiliations:** ^1^School of Economics and Management, Sanming University, Sanming, China; ^2^School of Accounting and Auditing, Guangxi University of Finance and Economics, Nanning, China

**Keywords:** early warning mechanism, major public health emergencies, poverty alleviation and marginal populations, risk matrix, risk prevention and control

## Abstract

Poverty alleviation is critical for sustainable development. Establishing a major public health emergency warning and prevention mechanism for poverty alleviation and marginal populations can effectively determine the overall risk situation and primary risk components in diverse regions. It is conducive to formulate specific policies for risk prevention and control of public health emergencies to prevent the occurrence of poverty relapses. Expert evaluation method is used to grade the risk impact and risk probability. Combined with the Borda ordinal value method to rank the importance of risk factors, a judgment matrix is constructed. The Analytic Hierarchy Process method is used to determine the weight of the risk module. And the impact risk of major health emergencies on poverty alleviation and marginal populations is comprehensively evaluated based on the quantified value of the risk impact level. The results indicate that the production and operation risks and unemployment risks in the case area have a relatively significant impact on poverty alleviation and marginal populations. Different regional government forces and regional economic development characteristics may also form different risk weights. Therefore, risk warning models should match government development and regional development goals and be established based on the characteristics of different regions. Effective risk control mechanisms and policies can only be developed based on the characteristics and behavioral traits of different populations in the region.

## 1 Introduction

Poverty is a global challenge that is affecting national prosperity and individual well-being. China has achieved remarkable outcomes in poverty alleviation. By the end of 2020, all 98.99 million Chinese rural people have been lifted out of poverty. However, it is still easy for the unstable population in poverty alleviation to fall back into poverty when they face the potential risks, such as major public health emergencies. It has become an important issue to consolidate the existing poverty alleviation achievements under unpredictable risks and gradually eliminate the obstacles of adverse factors, minimize the loss of poverty alleviation and marginal population, and reshape their vision for a better life in the future.

Public health emergencies not only directly cause damage to the public’s bodies and seriously threaten their lives, but also make the public feel fear and anxiety, and cause serious economic losses to countries and individuals. Consequently, all countries and regions attach great importance to the prevention and control of major public health emergencies. In order to prevent the occurrence of public health emergencies and reduce the harm they cause, Fujian Province has issued a series of policies and implemented several effective measures. These measures and policies aim to guide and regulate the emergency response efforts for various public health emergencies in Fujian Province. They also contribute to the effective prevention, timely control, and elimination of public health emergencies, and minimize their harms and impacts on society. In practice, these measures not only ensure the physical and mental health and life safety of the public, but also ensure that all medical and health rescue work is carried out quickly, efficiently, and orderly after the occurrence of public emergencies. By 2022, Fujian Province has established an emergency response system for public health incidents with a complete system, clear functions, efficient operation, and strong guarantees. The capacity of regular response to major public health emergencies and the medical rescue level of the health department toward various public emergencies have been significantly improved. How can one effectively implement the early warning, control, and guarantee of significant public health emergencies? Setting standards for early warning at the beginning of major public health emergencies and formulating control and action plans in order to form an integrated risk prevention and emergency guarantee mechanism is an important task for epidemic prevention and control and consolidating the results of poverty alleviation. Therefore, this study uses the Fujian Province as an example to build an emergency response management system for major public health emergencies for key monitoring targets such as unstable poverty alleviation households and marginal households. It establishes a comprehensive workflow from early warning identification to process control and guarantee mechanisms, in order to provide important policy ideas for greatly improving the anti-risk ability of key monitoring and assistance targets to cope with major public health emergencies.

## 2 Literature review

### 2.1 Research on poverty alleviation and marginalized populations returning to poverty

#### 2.1.1 Research on the theoretical basis of returning to poverty

Amartya Sen effectively analyzes from the perspective of welfare economics that the insufficient ability of poverty-stricken populations lies in the absence of fairness and justice in feasible capabilities (1). Scholars represented by Sen believe that improving feasible capabilities should prioritize the social justice and fairness of vulnerable groups in the face of disasters (2). They are well aware of the catastrophic impact of individual vulnerability and risk invasion on the poverty-stricken population, which is falling into poverty (3). Eliminating the differences in opportunities, resources, and rights when facing risks has become the foundation for setting early warnings, developing controllable plans, rapidly implementing plans, and establishing reasonable safeguard measures for poverty-stricken populations in the event of emergencies (4). Therefore, it is necessary to explore the procedures and controls for dealing with emergencies faced by poverty alleviation and marginalized populations from this perspective.

#### 2.1.2 Research on the reasons for the return to poverty of poverty alleviation and marginalized populations due to sudden events

The absence of personal abilities and the marginalization of communication skills have led to the isolation of poverty alleviation and marginalized populations in information acquisition and transportation (5, 6). It was difficult for them to overcome their already fragile coping abilities in emergency situations. Although poverty alleviation and marginalized populations have moved away from absolute poverty, their vulnerability and marginalization issues have not been fundamentally resolved, and there are always some risks of returning to poverty in the event of emergencies (7). Researchers have found that although vulnerability and marginalization cannot be identified as direct causes of poverty among poverty alleviation and marginalized populations in the event of sudden events, their interweaving increases the probability of poverty relapse risk (8, 9). Through the analysis of vulnerability and marginalization, researchers have revealed that the essential reason why poverty alleviation and marginalized populations return to poverty due to emergencies is the lack of accumulated resources and the ability to create and acquire various resources, which makes it difficult to maintain the damage caused by emergencies (10). Correspondingly, these infringements have increased the degree of psychological damage to poverty alleviation and marginalized populations (11). According to Polsky’s “Exposure-Sensitivity-Adaptability” framework, impoverished and marginalized populations are overly dependent on social assistance and incapable of coping with external risk factors. Furthermore, poverty alleviation and marginalized populations overly rely on social assistance and fail to respond to external risk factors. Therefore, it is difficult for them to form appropriate adaptability when unexpected events occur, and it is inevitable that they will fall into the abyss of poverty (12).

#### 2.1.3 Research on measures to prevent return to poverty

Many researchers currently believe that the best way to eradicate poverty and prevent returning to poverty is to promote industries to reduce the damage caused by risks by enhancing their ability (13, 14). Meanwhile, researchers point out that the goal of eliminating poverty can also be achieved through improving education and encouraging entrepreneurship (15). With the continuous advancement of research and practice, researchers have realized that it is difficult to achieve consistency from individual to overall planning solely through external assistance. Starting from individual risk prevention, reducing the probability of returning to poverty has become the mainstream of current research (16). Building a mechanism for early warning of poverty relapse and using big data and other methods to prevent the occurrence of poverty relapse has become a novel breakthrough in current research concepts (17). Based on the theory of sustainable livelihoods, risk assessment is constructed as the starting point, with a focus on controlling and monitoring risk factors. Only by enhancing the channels and capabilities of poverty alleviation and marginalized populations in resource supply and information exchange, it can effectively prevent a return to poverty (18). The above measures are not only aimed at providing relief and assistance to organizations and society, but also at enhancing the ability of poverty alleviation and marginalized populations to cope with major public health emergencies through comprehensive measures such as economic accumulation, social governance, and public service quality, which strengthens their ability to resist risks (19, 20).

### 2.2 Research on emergency management of public health emergencies

The 1918 influenza pandemic proved to be an important turning point in the study of major health events, as researchers focused on its impact on future societies and neglected the effective consideration of individual safety (21, 22). The SARS incident in 2003 prompted emergency management research to pay more attention to individual lives and maintain social security. How to establish a timely early warning system, from the occurrence of incidents to risk identification to program setting and response measures, has been analyzed in detail (23). Nelson et al. (24) clarified the steps of emergency handling of public health emergencies into multiple stages such as monitoring, confirmation, warning, and disposal. Zhao et al. (25) studied the life cycle attributes of public health emergencies using Steven Fink’s four-stage model of transmission. From the root cause of the incident to the analysis of risk factors to the risk elimination process, and finally the concept of focusing on prevention and establishing a linkage mechanism to reduce risk damage was proposed (25). Wang Xiaoxiao et al. (26) believe that paying attention to basic risk management work, such as daily emergency preparedness and early warning, is an important foundation for emergency management. With the gradual promotion of governance thinking, Lin Weiwei (27) used governance theory to construct an overall idea of a ‘multi-object, multi-subject, multi-element, multi-tool, multi-stage’ health governance system. The aforementioned research did not comprehensively analyze the degree of risk impact, nor did it consider the process of risk control and disposal from the perspective of information collection, risk level discrimination, warning mechanism response, and dynamic feedback updates. It is difficult to comprehensively evaluate the impact of sudden public health emergencies on poverty alleviation and marginalized populations. Therefore, by taking into consideration the internal and external environment of poverty alleviation and marginalized populations and evaluating the risk factor set of major health emergencies from comprehensive factors such as industrial risks, unemployment risks, disease risks, assistance risks, and production risks, an effective early warning mechanism can be accurately established.

## 3 Methodology

The impact of major public health emergencies on poverty alleviation and marginalized populations stems from the external environment, within households, and within the poor themselves. This impact is long-lasting, multifaceted, and complex. The major public health emergency not only seriously affects the physical and mental health of the people but also impacts social and economic development. Therefore, when the public health emergencies occur, it is very necessary and urgent to strengthen the early warning, control, and emergency guarantee mechanisms for major public health emergencies.

### 3.1 The mechanism-building process

**Figure 1:**
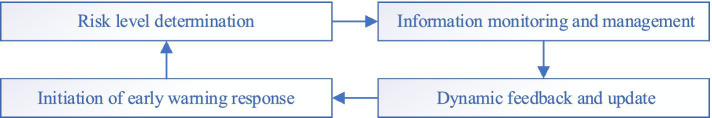
Risk early warning mechanism for major public health emergencies.

The risk early warning mechanism for major public health emergencies mainly consists of four aspects: information monitoring and management, risk level determination, initiation of early warning response, and dynamic feedback and update, forming a closed-loop full-cycle management model (see Figure 1). In practice, first of all, through the village cadres, surveys and the big data monitoring platform for rural poverty alleviation warning, the data of relevant departments are summarized and compared to find and identify farmers who meet the basic conditions of monitoring objects. Second, on the basis of information monitoring, the risk matrix method is employed to comprehensively evaluate the risk of major public health emergencies. Third, according to the monitoring information and risk judgment results of relevant departments, the risks are divided into different levels, and then different assistance measures are implemented. Finally, the joint departments regularly update the risk factors in the big data monitoring platform for early warning of returning to poverty and the database of risk elimination.

#### 3.1.1 Information monitoring and management

Major public health emergencies, such as major epidemics, are a dynamic development process. Therefore, real-time information collection and accurate monitoring of various data indicators are the basis for building early warning, risk control, and emergency support mechanisms. There are two main methods to collect information: village cadre visits, surveys, and big data analysis. The first step is to conduct regular visits and surveys of poverty alleviation households and marginal households by village cadres to understand the impact of major public health emergencies and collect authentic and reliable data and information. And then carry out information verification, investigation, and real-time tracking so as to fully ensure that no household or person is left behind, and do the basic job of prevention. The second step is for the government to establish a big data monitoring platform for rural poverty alleviation warning and improve the management of various indicator data of poverty alleviation and marginal households. According to the actual situation of each province or city, the monitoring indicators are mainly formulated in terms of annual household income, disposable income, labor salary, education, medical treatment, living conditions, and human capital. Grassroots workers will promptly input the data reported by each village and the information collected during regular visits into the database. The big data monitoring platform for early warning of rural poverty return and relevant departments such as medical care, education, and civil affairs have cooperated in an orderly manner in order to ensure the accuracy and objectivity of household information data collection. Combine big data and early warning systems of returning to poverty and use information technology to provide effective support for the establishment of an early warning system for rural return to poverty. To improve the accuracy and standardization of data collection, the following steps are required: First, establish standardized data collection processes. Develop unified data collection standards, questionnaires, and interview guidelines in order to ensure that all data collection personnel collect data according to the same standards and methods. Second, utilize advanced technological tools. Utilize mobile devices and professional software for data collection and preliminary analysis for reducing manual input errors and improving data processing efficiency. Third, provide training for data collection personnel. Regularly train data collection personnel on collection methods, data entry software operations, and data confidentiality education to ensure they have the necessary knowledge and skills. Finally, implement data quality control. Set up inspection and verification stages in data collection, input, and processing to ensure the accuracy of the data. For example, sampling and verifying the collected data are essential.

#### 3.1.2 Risk level determination

The risk factors arising from major public health emergencies are complex. In view of the multi-dimensional characteristics of the risk of major public health emergencies and the difficulty of quantitative evaluation, a combination of quantitative and qualitative risk measurement and evaluation methods was proposed. Through the methodological evaluation, ranking, and comparison process, it helps local governments and relevant departments to identify and clarify the risk degree of major public health emergencies and the impact on poverty alleviation and marginalized populations. According to the principle of multi-attribute grade evaluation, which was combined with the advantages of quantitative research methods such as the analytic hierarchy process and the Borda ordinal value, a risk matrix method was proposed to comprehensively assess the risk of major public health emergencies. First, the expert survey method is combined with modern fuzzy mathematics and statistical analysis tools to evaluate the level of risk impact degree and impact probability. The expert survey method, also known as the Delphi method, involves soliciting the opinions of experts on the problem to be predicted, organizing, summarizing, and counting them, then anonymously feeding them back to the various experts, soliciting their opinions again, focusing them again, and feeding them back again until a consensus is reached. Then, the importance of the impact risk is ranked by the Borda ordinal value method, and the judgment matrix is constructed. The Borda ordinal value method is mainly based on multiple risk evaluation criteria, through a certain calculation method to get the Borda number of each risk, and then according to the size of the Borda number of risks to get the corresponding Borda ordinal number, in order to realize the importance of the risk of the cross-category rank ordering. The calculation rules can significantly reduce the ambiguity of the risk level in the risk matrix, separate the relatively important risks from the collection of risks with the same risk level, and reduce human subjectivity. Finally, the analytical hierarchy method is used to determine the weight of the impact risk module and the overall impact risk level. Analytic hierarchy method is a systematic method based on the idea of hierarchy and level-by-level comparison. It decomposes the problem into multiple levels, compares the elements in each level two-by-two, and derives the weight ordering in order to optimize the decision-making as a goal and multiple options.

#### 3.1.3 Initiates an alert response

Based on the monitoring of real-time information and risk levels, corresponding to the risk level of major health emergencies on poverty alleviation and marginalized populations, form early warning mechanisms of different levels and take timely interventions to prevent the risk level from beginning. According to the analysis of the risk assessment model, the risk category and level of public health emergencies are studied and evaluated. Various emergency response plans and corresponding disposal methods are initiated according to factors such as severity, controllability, and impact scope. Adopt two modes of system automatic warning and personalized warning to set the warning line. If a warning is triggered, the system will promptly dispatch home inspection instructions. Automatic system warning refers to the warning platform setting warning parameters based on risk levels to classify and warn potential objects. For poverty alleviation households and marginalized households with high risk levels, deep degrees, and the wide impact of major public health emergencies, it requires rapid decision-making and timely assistance. Through on-site assessment and judgment of major public health emergencies, initiate early warning responses, and carry out relevant medical rescue and assistance activities. For moderate risks, a comprehensive investigation is also needed to determine whether they meet the conditions for inclusion in social assistance. Low-risk warning objects are generally dynamically tracked. Personalized early warning refers to giving early warning to families with particularly high single-risk. The government departments, such as medical, civil affairs, emergency departments, and other relative departments, share data every month in order to help the villages and towns grasp the information of returning to poverty and implement targeted assistance.

#### 3.1.4 Dynamic feedback updates

The evolution of major public health emergencies is changing constantly; there are various uncertainties, and the risks brought to unstable and marginalized households in poverty alleviation also show obvious characteristics of instability and easy fluctuations. After establishing its corresponding early warning mechanism, it is important to take the necessary measures to intervene, establish scientific and timely emergency measures, and use different intervention methods according to different risk factors to effectively control risks. Specifically, it includes the timely updating of risk factors for major public health emergencies and dynamically updating warning data for poverty relapse. The warning lines are usually adjusted annually based on economic and social development. Adjust and increase disposal measures in response to new emergencies to control risks. Villages and towns should undertake at least one risk elimination assessment every quarter. If the income and basic life are stable and the risk of returning to poverty is eliminated stably, or the risk of returning to poverty disappears naturally, they should be marked as “risk elimination.” Villages and towns should continue to focus on this issue and do a good job in the follow-up management of risk elimination. However, mechanism building is a complex and long, dynamic process. In order for the early warning mechanism to play a stable role, it is also necessary to continuously monitor in real time for a period of time during major public health emergencies, pay attention to the impact of the event on the monitoring target, continue feedback and evaluation, and take strengthening measures. At the same time, timely communication with grassroots workers to form effective bilateral interaction and constantly update the corresponding database according to the evolution of the incident can ensure the timeliness and effectiveness of emergency management of major public health emergencies.

In the above process, clarifying the possible risk factors of major public health emergencies on poverty alleviation and marginalized populations, finding their impact weight, and using scientific methods to evaluate and compare risks are important contents of constructing a comprehensive assessment model for risk early warning of major public health emergencies. Therefore, the next part mainly focuses on the construction of risk assessment and early warning models for major public health emergencies.

### 3.2 Risk early warning model construction

**Figure 2:**
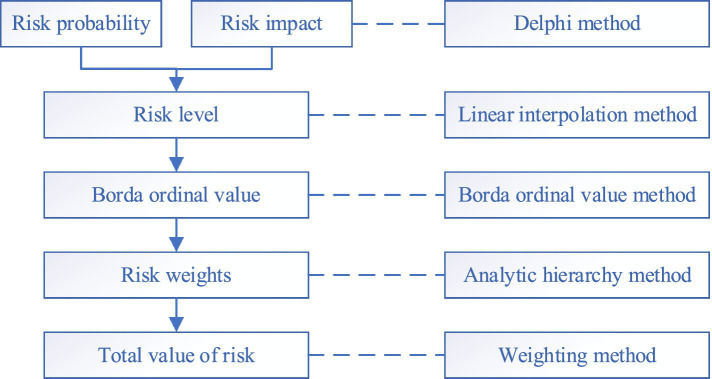
Risk matrix model.

An improved risk matrix is used to assess risks both qualitatively and quantitatively (see Figure 2). Based on the comparison of the analytic hierarchy process, the improved risk matrix is able to accurately distinguish the rationality of qualitative judgment by using the Borda ordinal value method. At the same time, through normalization, qualitative data and quantitative data are combined to find a unified trend of solving problems between them. By using the objectivity of quantification and the directionality of qualitative data, the rationality and reliability of the research can be achieved. First, it is necessary to identify the specific risks of major public health emergencies to poverty alleviation and marginalized populations. Second, since the risks are uncertain and difficult to predict, the Delphi method is selected to judge the probability and impact of different risks based on the experience of experts, and the quantitative values are obtained by referring to the rating standards. Third, comprehensively considering the probability of risk occurrence and risk impact, the linear interpolation method is used to calculate the quantitative value of each risk item to determine the risk level. Linear interpolation is a method of determining the value of an unknown quantity between two known quantities by connecting them with a straight line. This approach assumes that the development and changes of phenomena are linear and uniform, so linear interpolation can be performed using linear equations. And then, combined with the Borda ordinal value method and the analytic hierarchy method, the ranking and weight of risk items at all levels were determined to eliminate the risk knot and reduce the subjectivity of expert judgment. Finally, the weighting method is then used to calculate the total risk level value. After clarifying the overall risk level and ranking the importance of each risk, choose different risk reduction strategies, such as enhancing regional development capabilities, preventing and controlling disasters and emergencies, providing education and employment assistance, and improving the health status of vulnerable groups. The specific steps are as follows:

#### 3.2.1 Aggregation of risks

**Figure 3:**
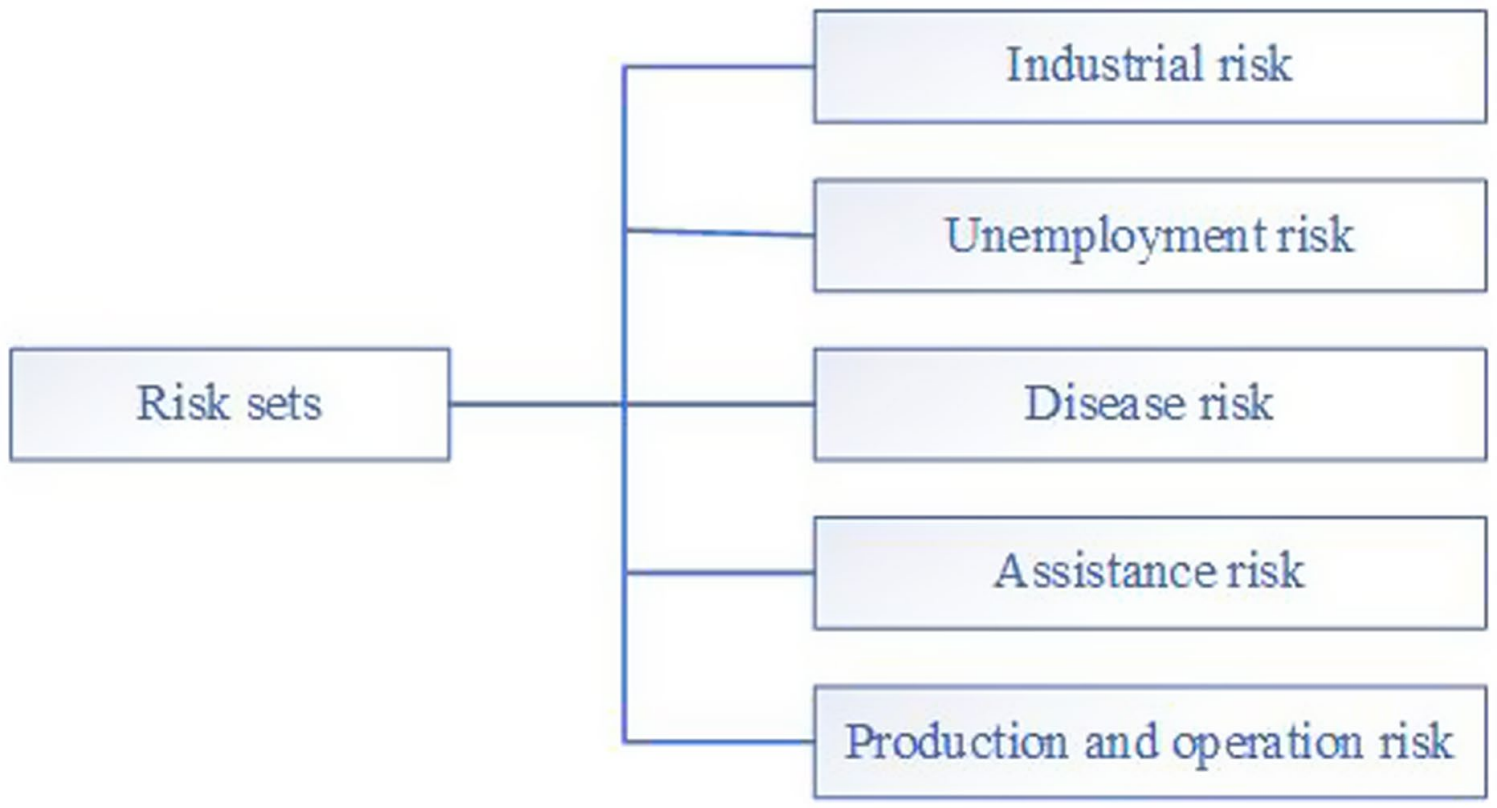
Risk factor set of major public health emergencies.

Affected by internal and external factors, the risks of major public health emergencies to poverty alleviation households and poor marginal households are multifaceted. Comprehensively and scientifically identifying the risks arising from major public health emergencies to poverty alleviation and unstable marginal households is an important basic work for effectively assessing, preventing, and controlling the risk of returning to poverty. It is also one of the important links in improving the emergency management mechanism for major public health emergencies. Through the results of investigation and analysis, the risks of major public health emergencies to unstable poverty alleviation households and marginal households will be comprehensively analyzed from five modules: industrial risk, unemployment risk, disease risk, assistance risk, and production and operation risk (see Figure 3). Industrial risks include the impact of major public health emergencies on the regional economy, poverty alleviation industries or certain industries, such as tourism, resulting in the reduction of income and living difficulties of unstable households and marginalized households. The term “unemployment risk” refers to the risk of a major public health emergency that causes unstable and marginalized households to lose their jobs and return to poverty without income sources. Disease risk refers to major public health emergencies, such as the novel coronavirus pneumonia epidemic, which cause unstable and marginal households to suffer from infectious diseases and mental health diseases such as panic due to their limited cognitive ability. Assistance risks include risks such as insufficient support personnel, lack of living materials, and short supply of medical supplies that may temporarily occur during major public health emergencies. Production and operation risks cover risks such as insufficient production materials, poor product sales, and insufficient production technical support that may occur in unstable and marginal households during major public health emergencies. Among these, industrial risk is the foundation. Disease risk is the direct cause of other risks deepening and increasing. The assistance risk depends on the level of unemployment risk and the strength of industrial risk, and production and operation risk is the key link of the total risk. Synthesize the above risk content to form a risk set.

#### 3.2.2 Determination of risk level

The level of risk depends on the risk probability and the degree of risk impact. Due to the complex and regional differences in the impact of major public health emergencies on unstable poverty alleviation households and marginalized households, it is difficult to quantitatively judge. So the Delphi method was used to analyze the degree of impact and probability of different risks based on the experience of experts and fuzzy mathematical methods. First, the risk impact level is divided into five levels: critical, serious, moderate, minor, and negligible, and the quantitative value range is defined (see Table 1). Second, divide the range of risk probabilities (see Table 2). Then, experts in the field are organized to give a reasonable estimate and score for the degree of influence and probability of occurrence of risk based on relevant information and previous experience. Finally, the average of the score values of each expert is used as the comprehensive evaluation value.

**Table 1:** Degree of risk impact.

Risk impact level	Risk impact level quantitative value range	Description
Critical	4<i≤5	Once risk events occur, poverty alleviation and marginalized populations are at great risk, and a large number of people will fall back into poverty.
Serious	3<i≤4	Once the risk event occurs, it will lead to a greater risk of poverty alleviation and marginalized populations falling back into poverty, which will seriously affect the effectiveness of poverty alleviation.
Moderate	2<i≤3	Once a risk event occurs, it will lead to certain difficulties in poverty alleviation and marginalized populations, which will affect the effectiveness of poverty alleviation to a certain extent.
Minor	1<i≤2	Once a risk event occurs, it will have a slight impact on poverty alleviation and marginalized populations, and the indicators of poverty alleviation effectiveness can still be guaranteed.
Negligible	0<i≤1	Once the risk event occurs, there is basically no impact on poverty alleviation and marginalized households.

**Table 2:** Explanation of risk probability.

Risk probability range (%)	Description
0–10	Very unlikely to happen
11–40	Basically impossible to happen
41–60	May happen
61–90	More likely
91–100	Most likely

Comprehensively considering the above two factors, a risk level comparison table is obtained to reasonably divide the risk level (see Table 3). According to the national standard of the People’s Republic of China, “Information Security Technology—Risk Assessment Specification for Information Security( GB/T20984−2007 ),” the risk level is divided into five levels: very low, low, medium, high, and very high, and the corresponding quantitative value range of the risk level is defined. The range of risk level can be obtained by checking the quantitative value of risk impact and risk probability determined by the Delphi method into the risk level comparison table.

**Table 3:** Risk level comparison table.

Risk PR range (%)	Risk implications									
	Negligible		Minor		General		Severe		Essential	
	Value	Level	Value	Level	Value	Level	Value	Level	Value	Level
0–10	0	VL	0–0.5	VL	0.5–1	VL	1–1.5	L	2–2.5	M
11–40	0	VL	0–0.5	VL	1–1.5	L	1.5–2	L	2.5–3	M
41–60	0–0.5	VL	0.5–1	VL	1.5–2	L	2–3	M	3–4	H
61–90	0–0.5	VL	1–1.5	L	2–2.5	M	3–3.5	H	4–4.5	VH
91–100	0.5–1	VL	1.5–2	L	2.5–3	M	3.5–4	H	4.5–5	VH

PR, probability; L, low; VL, very low; M, medium; H, high; VH, very high.

In order to obtain a definite quantitative value of risk level, a determined risk level value is calculated by linear interpolation. Suppose I is the quantitative value of the impact level of a certain risk, which belongs to the interval $\left[I_{1},I_{2}\right]$ . The probability of occurrence of this risk is P and belongs to the range $\left[RP_{1},RP_{2}\right]$ . The risk level is *RR* and the risk level range $\left[RR_{1},RR_{2}\right]$ can be found in the risk level comparison table (Table 3) according to the risk impact level and risk probability. The risk level calculation formula is shown in Equation 1.


(1) $$RR=RR_{1}+\frac{\left(I-I_{1}\right)\left(P-P_{1}\right)}{\left(I_{2}-I_{1}\right)\left(P_{2}-P_{1}\right)}\left(RR_{2}-RR_{1}\right)$$

#### 3.2.3 Determination of risk weights

##### 3.2.3.1 The Borda ordinal value calculation

When there are many risk factors, there will be two or more risk factors at the same risk level, that is, the risk knot, which is not conducive to comparing risk items, and it is difficult to judge the size of the risk. On the basis of the risk matrix method, the Borda ordinal value method is used to rank risk factors of different risk levels by calculating the ordinal values of different risk items, so as to reduce the risk knot to a certain extent and evaluate the risk size more accurately.

The Borda number of risk i can be calculated from Equation 2:


(2) $$b_{i}=\overset{3}{\underset{k=1}{\sum }}\left(N-r_{ik}\right)$$

In formula 2, N is the number of risk factors, k is a criterion, where k=1 represents the risk probability criterion, k=2 represents the risk impact criterion, k=3 represents the risk level criterion, and $r_{ik}$ represents the risk level of risk i under the criterion k . Compare the Borda number of each risk factor to rank the risk level according to different criteria.

##### 3.2.3.2 Use analytic hierarchy method to calculate the risk weights and perform one-time testing

The degree of influence of each risk item on the risk of major public health emergencies is different, and the analytic hierarchy method is used to give corresponding weight to the importance of each indicator. In order to reduce the influence of subjective judgment of different evaluators, the Borda ordinal value ranking results are used as the basis for expert scoring in the analytic hierarchy method, which reduces subjectivity and makes the calculation results of risk weights more scientific.

First, the target layer, criterion layer, and indicator layer of the analytic hierarchy structure are built according to the risk factor set. Then, the experts compare the elements of each layer in pairs, combined with the 1–9 scale method (see Table 4), score the relative importance of each factor in each layer, and construct a judgment matrix.

**Table 4:** Scale and meaning of 1–9.

Scale	Meaning
1	Risk i is equally important as risk j
3	Risk i is slightly more important than risk j
5	Risk i is generally more important than risk j
7	Risk i is significantly more important than risk j
9	Risk i is more important than risk j
2,4,6,8	The median of the two adjacent scales above
Reciprocal	The comparative value of risks j and i

The maximum eigenvalue $\lambda_{\text{max}}$ and its eigenvectors of each judgment matrix are calculated and normalized to obtain the ranking weights of each risk (Equation 3). Calculate the consistency index CI, average random consistency index RI, and random one-time ratio CR for one-time testing (Equations 4–6). If CI is closer to 0, the higher the consistency satisfaction; conversely, the degree of inconsistency is high. Considering the inconsistency caused by random factors, it is also necessary to determine the test coefficient CR. If CR < 0.1, it means that the judgment matrix passes the one-time test. Otherwise, if it fails, the judgment matrix needs to be adjusted.


(3) $$\lambda_{\text{max}}=\frac{1}{n}\overset{n}{\underset{i=1}{\sum }}\frac{{\left(\mathrm{Aw}\right)}_{i}}{w_{i}}$$

(4) $$CI=\frac{\lambda_{\text{max}}-n}{n-1}$$

(5) $$RI=\sum \frac{CI_{n}}{n}$$

(6) $$CR=\frac{CI}{RI}$$

The relative importance weights of the risk indicators at the lower level and the risk indicators at the previous level are calculated layer by layer from the criterion layer to the indicator layer, and the weights are sorted and tested at one time.

#### 3.2.4 Calculation of the total value of risk

The quantitative value of the total risk level can be calculated using the weighting method (Equation 7). The total risk value TRR can be obtained by multiplying the grade $RR_{n}$ of each sub-risk item by its corresponding risk weight $W_{n}$ _,_ respectively. The risk level is determined according to the risk level corresponding to the TRR . In the same way, the risk level of each indicator at the criterion layer can be calculated, the risk level of each risk item of major public health emergencies can be obtained, and targeted risk prevention and control measures can be proposed.


(7) $$TRR=\underset{n=1}{\sum }RR_{n}\times W_{n}$$

In summary, this section outlines the process of constructing a risk warning mechanism and risk warning model for major public health emergencies. Next, we will apply it to practical cases for verification and explanation.

## 4 Case study of risk early warning assessment

This section obtains first-hand data through questionnaire surveys and on-site interviews, which provides a reliable basis for empirical research on the impact risk assessment of major public health emergencies and further verifies the above-mentioned comprehensive risk early warning assessment model. The authentic situation of poverty alleviation and marginal population in S City, Fujian Province, was selected, and the actual numerical simulation was conducted. The implementation steps of the risk early warning assessment model were explained and explained, and the effectiveness of the model was verified.

### 4.1 Case brief

S City, Fujian Province, has made numerous innovations and attempts to target poverty alleviation. Since the outbreak of COVID-19, S City has made every effort to overcome the impact of the epidemic by helping poor people to stabilize employment, promote production, and strengthen security. This will help promote poverty alleviation and a well-off society. During the period, S City carried out a number of measures and established a “daily tracking and weekly summary” supervision mechanism to timely grasp the impact of the epidemic on poor objects. A total of 208 households with 753 people who may return to poverty due to factors such as the epidemic will be monitored and managed separately with targeted assistance. The “one-click poverty report” online declaration system and poverty alleviation service hotline have been opened, and poor households can apply for monitoring targets if they have the risk of falling back into poverty due to the sudden drop in income and sudden increase in expenditure caused by the impact of the new crown epidemic. According to the statistical data provided by the Municipal Bureau of Agriculture and Rural Affairs of S city, in 2020, all 12,855 poor households affected by the epidemic were employed, 197 poor households in industrial development developed production, 18 poverty alleviation workshops have resumed production and work, and 452 poverty alleviation projects have been started.

After alleviating poverty, S City still actively monitors and helps unstable and marginalized households in poverty alleviation and constantly consolidates and expands the achievements in poverty alleviation. For households that have been lifted out of poverty but are unstable, on the basis of enjoying various policies, strengthen assistance according to the actual situation.

### 4.2 Numerical simulation

Based on the analysis of the risk early warning model of major public health emergencies and the research of S city in Fujian Province, taking S city as an example, relevant data were obtained through interviews, questionnaires, telephone and network consultations, etc., and numerical model simulation was carried out to illustrate the application of the risk early warning assessment model.

#### 4.2.1 The selection of the risk set

In order to verify the risk early warning assessment model more accurately and effectively and evaluate the risks of major public health emergencies to poverty alleviation and marginalized populations, through the investigation of various cities in Fujian Province, combined with the actual situation of S City, and on the basis of reasonable judgment by experts from relevant agricultural and rural departments at all levels with long-term accumulated experience, the risks brought by major public health emergencies to the unstable population and marginalized population of poverty alleviation are grouped into five factors: industrial risk, unemployment risk, disease risk, assistance risk, and production and operation risk (see Figure 3 for details).

#### 4.2.2 Determination of risk levels

Questionnaires and interviews were used to obtain the quantitative value of the risk impact of major public health emergencies on poverty alleviation and marginal populations in Tables 1, 2, along with the original assessment data of risk occurrence probability. The subjects of the questionnaire survey and interviews mainly include experts from the emergency management department of major public health emergencies in Fujian Province, experts from relevant departments of agriculture and rural affairs, research experts in this field, as well as relevant departments at all levels of city, county, township, and village, streets, communities, village cadres, and some unstable and marginal households that have been lifted out of poverty. A total of 148 questionnaires were collected from the survey. First of all, the respondents made reasonable judgments based on their own experience and relevant information and scored the quantitative value of risk impact and the probability of risk occurrence. Second, the simple arithmetic mean method was used to process the quantified value of the risk impact and the risk occurrence probability of the five risk factors, respectively, and the mean values were taken. The results obtained after processing are as follows: the quantitative value of the risk impact of the five major risks of industrial risk, unemployment risk, disease risk, assistance risk, and production and operation risk brought by major public health emergencies to poverty alleviation and marginalized population in S city was, $\left\{2.97\;2.97\;2.82\;2.52\;2.98\right\}$ , and the probability of risk occurrence was $\left\{\begin{array}{ccccc} 48.51\% & 49.91\% & 47.93\% & 42.27\% & 50.02\% \end{array}\right\}$ , respectively, and the quantitative value of risk impact and risk occurrence probability were filled in the corresponding columns in Table 5.

**Table 5:** Risk matrix.

Risk items	Risk probability P	Quantitative value of risk impact I	Risk level (*RR*)		Borda ordinal value	Risk weights *W*
			Value	Level		
Industrial risk	48.51%	2.97	2.38	Medium	2	0.195
Unemployment risk	49.91%	2.97	2.45	Medium	1	0.320
Disease risk	47.93%	2.82	2.30	Medium	3	0.119
Assistance risk	42.27%	2.52	2.03	Medium	4	0.075
Operation risk	50.02%	2.98	2.47	Medium	0	0.292

Furthermore, according to the linear interpolation method (Equation 1) and the risk level comparison table (Table 3), the risk levels of the five major risk factors are calculated, respectively, and filled in Table 5. The specific calculation is as follows:


$$\begin{array}{l} {RR}_{\mathrm{industry}}=2+\frac{(2\mathrm{.97-2)*(48.51-41)}}{(\mathrm{3-2)*(60-41)}}(\mathrm{3-2)=2}.38\\ {RR}_{\mathrm{unemployment}}=2+\frac{(2\mathrm{.97-2)*(49.91-41)}}{(\mathrm{3-2)*(60-41)}}(\mathrm{3-2)=2}.45\\ {RR}_{\mathrm{disease}}=2+\frac{(2\mathrm{.82-2)*(47.93-41)}}{(\mathrm{3-2)*(60-41)}}(\mathrm{3-2)=2}.30\\ {RR}_{\mathrm{assistances}}=2+\frac{(2\mathrm{.52-2)*(42.27-41)}}{(\mathrm{3-2)*(60-41)}}(\mathrm{3-2)=2}.03\\ {RR}_{\mathrm{operation}}=2+\frac{(2\mathrm{.98-2)*(50.02-41)}}{(\mathrm{3-2)*(60-41)}}(\mathrm{3-2)=2}.47 \end{array}$$

#### 4.2.3 Determination of Borda ordinal values and risk weights

*Step 1*. The Borda ordinal value method was applied to rank the importance of each risk factor. Apply Equation 2 to calculate the Borda number of 5 risk items, respectively. Then determine the Borda ordinal value of each risk item by comparing the Borda number with other risk items. The specific calculation is as follows:


$$\begin{array}{l} b_{\mathrm{industry}}=\left(5+1\right)+\left(5+2\right)=7\\ b_{\mathrm{unemployment}}=\left(5+1\right)+\left(5+1\right)=9\\ b_{\mathrm{disease}}=\left(5+3\right)+\left(5+3\right)=4\\ b_{\mathrm{assistances}}=\left(5+4\right)+\left(5+4\right)=2\\ b_{\mathrm{operation}}=\left(5+0\right)+\left(5+0\right)=10 \end{array}$$

Combined with the Borda number for each risk item, the Borda ordinal values of industrial risk, unemployment risk, disease risk, assistance risk, and production and operation risk can be determined as follows: $\left\{\begin{array}{ccccc} 2 & 1 & 3 & 4 & 0 \end{array}\right\}$ . Therefore, it is concluded that the importance of the impact of major public health emergencies on poverty alleviation and marginalized populations in S City is ranked as production and operation risk, unemployment risk, industrial risk, disease risk, and assistance risk.

*Step 2*. Determines the weights for each risk factor based on the Borda ordinal value. According to the Borda ordinal values obtained in step 1, experts are invited to further judge and correct, and a judgment matrix A is constructed on the basis of a pairwise comparison of the five risk factors according to their degree of importance.


$$A=\left(\begin{array}{ccccc} 1 & \frac{1}{2} & 2 & 3 & \frac{1}{3}\\ 2 & 1 & 3 & 4 & \frac{1}{2}\\ \frac{1}{2} & \frac{1}{3} & 1 & 2 & \frac{1}{4}\\ \frac{1}{3} & \frac{1}{4} & \frac{1}{2} & 1 & \frac{1}{5}\\ 3 & 2 & \frac{1}{4} & 5 & 1 \end{array}\right)$$

Apply analytic hierarchy to determine the weight of each risk factor. Through the calculation of Matlab, the consistency index CI = 0.005 and the random consistency ratio CR = 0.004 < 0.10, the calculation results of the judgment matrix A show consistency, indicating that the matrix judgment evaluation is reasonable. The weights of the five risk items, $W=\left\{\begin{array}{ccccc} 0.195 & 0.32 & 0.119 & 0.075 & 0.292 \end{array}\right\}$ , are filled in Table 5.

#### 4.2.4 Comprehensive risk assessment

Based on the above calculation results, specific data such as the quantitative value of risk impact level, probability of occurrence, quantitative value of risk level, and risk weight are filled in into the risk matrix (Table 5).

The values in Table 5 are substituted into Equation 7 to calculate the TRR of poverty alleviation and marginalized population risk level of major public health emergencies in S City.


$$\begin{array}{l} TRR=\overset{5}{\underset{n=1}{\sum }}RR_{n}\times W_{n}=2.38^{*}0.195+2.45^{*}0.320+2.30^{*}0.119+\\ 2.03^{*}0.075+2.47^{*}0.292=2.40 \end{array}$$

2.40 is in the range [2,3], so it is a moderate risk.

## 5 Results

In summary, we can get:

First, the risk level of poverty alleviation and marginalized population of major public health emergencies in S City is medium. The medium risk means that once a risk event occurs, it will lead to certain difficulties in poverty alleviation and marginalized populations. This will affect the effectiveness of poverty alleviation to a certain extent. Especially, according to the above empirical analysis results, the S city has a higher risk of unemployment and weaker production and operation capabilities. S City, which is geographically isolated, has deficiencies in industrial development and the adaptation of the population to the skills required by the industry. Therefore, it is important to strengthen ties with advanced regions. It is also necessary to upgrade the industrial structure, consolidate the material foundation and cooperation foundation of industrial development, and enhance the ability of the marginal population to get rid of poverty so as to significantly reduce industry risks and unemployment risks and prevent the increase of the probability of returning to poverty.

Second, according to the Borda ordinal value method, the risks brought by major public health emergencies to poverty alleviation and marginalized populations in S City are ranked as production and operation risks, unemployment risks, industrial risks, disease risks, and assistance risks. This result provides a certain theoretical reference and decision-making basis for the public on emergency management and poverty governance of major public health emergencies in S City. Targeted measures can be taken in advance to reduce the impact of risks according to the importance of various risks before major public health emergencies occur.

## 6 Discussion

Improving the quality of life of the people who have been lifted out of poverty and allowing them to fully reap the benefits of human development has become an important focus of many scholars to carry out research on consolidating poverty alleviation. This study conducts an in-depth analysis and empirical research into the early warning and control of the risk encountered by poverty alleviation and marginalized populations in the major public health emergencies. It also contributes to the research field of consolidating the achievements of poverty alleviation, with the following theoretical implications and practical value.

### 6.1 Theoretical implications

Through the above-mentioned empirical research, compared with the general population, poverty alleviation and marginalized populations have problems such as insufficient industrial scale, weak ability to cope with risks, low degree of disease resistance, difficulty in ensuring the sustainability of assistance, and weak resistance to market risks, resulting in a situation that is difficult to cope with sudden risks. How to reduce poverty alleviation and the loss of marginalized people according to the logic of risk identification, risk control, and emergency support has become an important measure to effectively consolidate the achievements of poverty alleviation.

By analyzing the environmental risks, self-capability risks, and social relief risks faced by poverty alleviation and marginalized populations, this study explores the essence of the special risks experienced by vulnerable groups in society, that is, the singularity blind spot where public choice and public relief theories need to be perfected. There is still a long way to go to consolidate poverty alleviation in China. Starting from the proposal of risk factors, risk identification, risk control, and safeguard measures, reducing the blindness of public choice and public relief theory, and consolidating the key areas and key points of public choice from the vulnerable points of poverty alleviation and marginalized populations, only then can we continuously improve the protection mechanism for vulnerable groups. At the same time, the empirical research in this study also shows the difference in the degree of risk impact of different factors in different regions and uses utility theory as a guide to provide early warning and key work deployment by ranking. This provides a theoretical basis for the implementation of public relief weight arrangements.

Compared to general risk theory, the risk mechanism of special groups in specific situations should focus more on the autonomy, self-ability, and environmental impact of the population, as well as the relationship and degree of maintenance with other groups. Therefore, the establishment of the risk model takes into account the ability level of poverty alleviation and the marginalized population itself, environmental factors, and the degree of assistance of other groups, which further enriches the risk-influencing factors formed by behavioral, psychological, and relationship connections. This is conducive to further reducing the uncertainty loss caused by inaccurate risk identification.

From the above theoretical interpretation, it is clear that the fundamental solution to the problem of poverty alleviation and marginalized populations returning to poverty due to public health emergencies lies in the reasonable coordination and arrangement among stakeholders represented by the government. Using the self-assessed capabilities of poverty alleviation and marginalized populations as the starting point for formulating government policies can not only effectively reduce the risk losses faced by individual poverty alleviation and marginalized populations, but also increase the efficiency of government policy implementation based on accurate data. Establishing information channels for poverty alleviation and marginalized populations, using intelligent means to obtain massive data, makes it easier to reduce the risk of returning to poverty.

### 6.2 Practical value

Practice testing is an important component of ensuring the authenticity and popularity of theory. At present, the support for poverty alleviation and marginalized people is based on working methods such as assistance from all sectors of society, self-help for poverty alleviation and marginalized populations, and comprehensive protection for vulnerable groups who are unable to do so. However, in different contexts, poverty alleviation and marginalized population work priorities and workflow design still lack efficient work ideas. This study implements practical and feasible logical ideas in risk identification, establishment, control, and assurance. In addition, this study takes S city in Fujian as a case study to provide practical and feasible logical thinking in risk identification, establishment, control, and protection. Compared to general areas, the terrain in the mountainous areas of S city makes it difficult for poverty alleviation and marginalized populations to obtain timely assistance such as materials and social support when they are at risk of major health emergencies. This will hinder the efficiency of keeping poverty alleviation and marginalized populations away from risks. Therefore, the following aspects are proposed in order to improve the risk of returning to poverty for poverty alleviation and marginalized groups in specific regions.

First, on the basis of identifying overall risk factors, an identification and control mechanism has been established. Through the above empirical research methods, by identifying individual factors, environmental factors, and group connection factors, the importance of risks similar to those in S city can be identified. It has an important guiding role in identifying the key points of the local society’s work.

Second, the calculation and construction of risk weights can quickly establish the key control scope of the safeguard mechanism. Through in-depth interviews with front-line workers and relevant departments, on the basis of the questionnaire, the factors that are not quantitative in nature are quantified, and the risk management and control priorities of different regions are reflected more intuitively, comprehensively, and realistically, laying the foundation for the formation of a fruitful work system.

Third, it provides a basis for formulating policies for preventing risks in poverty alleviation and marginalized population areas. When combined with the aforementioned risk matrix construction and calculation process, the overall risk situation and main risk composition of different regions can be judged, which is conducive to the formulation of specific policies for risk prevention. As a region characterized by mountains and hills, S city faces significant challenges due to poor transportation and difficulties in delivering goods. These challenges contribute significantly to the high levels of production and operation risk and unemployment risk in the area. To mitigate these risks, the government should devise effective policies focused on industrial linkage mechanisms and individual mutual assistance, aimed at preventing the escalation and spread of these risks.

## 7 Limitations

Although a series of measures have been proposed to improve the standardization of data collection, in practice, data collection may still be affected by the following situations: Monitoring and early warning of poverty alleviation typically require the integration of data from multiple sources. These data not only originate from diverse individuals, but also from diverse regions, and are influenced by both individual factors and regional differences. Specifically, in terms of individuals, there are certain differences in data collection due to various factors such as local culture and personal cognitive abilities. The regional impact is due to the differences in policies, economic development, and organizational construction among different regions, which lead to certain biases in the data. In addition, the influence of the quality of technical personnel in different regions and the differences in equipment also affect the overall quality of the data. Meanwhile, differences in the work attitude, knowledge, and professional abilities of data collectors can lead to differences in data quality.

To alleviate the limitations mentioned above, future studies will focus on the direction of data accuracy and deep-mine the data.

First, conduct psychological questionnaire surveys to examine poverty alleviation and marginalized populations. Based on a complete understanding of individual differences, the basic idea of psychological training should be formed. Through psychological training, capture the true information of individual risk data and use it to organize and deep-mine data.

Second, in response to regional differences, a third-party evaluation agency should be selected to assess the impact of economic policies and development differences with a fair attitude and mindset. Furthermore, the third-party evaluation agency should use the standardized processing tools to ensure that data subjectivity issues are controlled within a reasonable range.

Third, search for regions similar to the research sample for the full process of data input and operation. Explore the specific factors that contribute to data differences, and reduce the deviation of individual and regional data on the basis of controlling factors, thereby making the research conclusions more accurate.

## Data Availability

The original contributions presented in the study are included in the article/supplementary material; further inquiries can be directed to the corresponding author.
